# Conformational Ensembles Explored Dynamically from Disordered Peptides Targeting Chemokine Receptor CXCR4

**DOI:** 10.3390/ijms160612159

**Published:** 2015-05-28

**Authors:** Marian Vincenzi, Susan Costantini, Stefania Scala, Diego Tesauro, Antonella Accardo, Marilisa Leone, Giovanni Colonna, Jean Guillon, Luigi Portella, Anna Maria Trotta, Luisa Ronga, Filomena Rossi

**Affiliations:** 1 Department of Pharmacy University of Naples “Federico II”, and CIRPeB, Via Mezzocannone 16, I-80134 Naples, Italy; E-Mails: marian.vincenzi@unina.it (M.V.); diego.tesauro@unina.it (D.T.); antonella.accardo@unina.it (A.A.); 2 Institute of Biostructures and Bioimaging (IBB), National Research Council (CNR), Via Mezzocannone 16, I-80134 Naples, Italy; E-Mail: marilisa.leone@cnr.it; 3 Oncology Research Center of Mercogliano (CROM), Istituto Nazionale Tumori “Fondazione G. Pascale”, IRCCS, I-80131 Napoli, Italy; E-Mail: s.costantini@istitutotumori.na.it; 4 Molecular Immunology and Immuneregulation, Istituto Nazionale Tumori “Fondazione G. Pascale”, IRCCS, I-80131 Napoli, Italy; E-Mails: s.scala@istitutotumori.na.it (S.S.); portella@gmail.com (L.P.); amt78@libero.it (A.M.T.); 5 Center of Medical Informatics, AOU, Second University of Naples, I-80138 Napoli, Italy; E-Mail: giovanni.colonna@unina2.it; 6 UFR des Sciences Pharmaceutiques—Collège Sciences de la Santé, INSERM U869, Laboratoire ARNA, Université de Bordeaux, 146 rue Léo Saignat, 33076 Bordeaux cedex, France; E-Mail: jean.guillon@u-bordeaux.fr

**Keywords:** intrinsically disordered protein (IDP), intrinsically disordered region (IDR), conformational ensemble, MD, NMR, CD, chemokine

## Abstract

This work reports on the design and the synthesis of two short linear peptides both containing a few amino acids with disorder propensity and an allylic ester group at the *C*-terminal end. Their structural properties were firstly analyzed by means of experimental techniques in solution such as CD and NMR methods that highlighted peptide flexibility. These results were further confirmed by MD simulations that demonstrated the ability of the peptides to assume conformational ensembles. They revealed a network of transient and dynamic H-bonds and interactions with water molecules. Binding assays with a well-known drug-target, *i.e.*, the CXCR4 receptor, were also carried out in an attempt to verify their biological function and the possibility to use the assays to develop new specific targets for CXCR4. Moreover, our data indicate that these peptides represent useful tools for molecular recognition processes in which a flexible conformation is required in order to obtain an interaction with a specific target.

## 1. Introduction

In the last decade, several proteins with native disordered structure—named Intrinsically Disordered Proteins (IDPs)—were reported to be involved in numerous key biological processes, including cell cycle control, regulation, signaling and binding events involving biological macromolecules [1,2,3,4,5]. Intrinsic disorder depends on the type of amino acids composing a certain protein [6,7,8]. The structural plasticity of proteins is mainly induced by the presence of residues inherently flexible, polar and charged. Through inherent flexibility, the mentioned residues have the ability to recognize different ligands, including other proteins, membranes, and nucleic acids [8]. Further studies have also shown that a flexible intrinsically disordered region (IDR) in a given protein can fold in several ways as a consequence of its binding to distinct partners. Moreover, the residue flexibility may also allow different protein sequences to recognize the same binding site on a specific partner [9]. Often, IDPs or IDRs are critically involved in receptor signaling, where they modulate the native and functional state of many proteins [10]. However, there is a limit to the understanding of the molecular mechanisms that regulate biological functions in which IDPs are involved: there is a lack of knowledge about the physical basis on which these processes rely [11]. Recently, authors have demonstrated that each IDP dynamically explores an ensemble of unfolded configurations, then each IDP assumes more stable and ordered structures after binding to their ligands is complete [12,13]. Molecular dynamics (MD) simulations can provide useful information about these issues in the spatio-temporal resolution. In fact, the flexibility of a protein can be derived from the trajectory of a MD simulation by calculating the root mean-squared fluctuations (RMSFs) of single atoms after removing the translational and rotational movements. Vibrations around equilibrium are not random but depend on local structure flexibility. Hence, RMSFs capture the fluctuation of each atom around average positions. Usually, the analysis of the average atomic mobility of backbone atoms (N, C^α^ and C atoms) during MD simulation gives insight into the flexible and rigid regions of proteins [14].

In this paper we designed short peptides as IDRs model systems in order to better understand molecular recognition processes involving IDPs. In particular, we focused on the two following synthetic linear peptides: PepK: Y-G-E-C-P-C-K-OAllyl; PepE: Y-G-E-C-P-C-E-OAllyl.

Both these peptides were designed by choosing a few residues with disorder propensity. At the *C*-termini of PepK and PepE a Lys and Glu residues were added, respectively, aiming to study the effect of charges in modulating the binding properties on a general target. The reactive allylic ester group was added to easily allow further chemical modifications finalized to improve biological activities.

The conformational behavior of the two peptides was firstly analyzed by CD (Circular Dichroism) and 1D [^1^H] and 2D [^1^H, ^1^H] NMR (Nuclear Magnetic Resonance) spectroscopies in order to establish their high flexibility and their inability to adopt a single ordered conformation. In addition, MD simulations indicated that the two peptides could be adequately represented by conformational ensembles.

In an attempt to define a biological significance for the above mentioned peptides, we have investigated their role as interactors of a known drug-target, *i.e.*, the CXCR4 receptor. Its overexpression results in metastatic dissemination of breast cancer cells to the lungs and lymph nodes and they contribute to melanoma tumor cell dissemination selectively to the lungs but not to the lymph nodes [15,16]. Moreover, CXCR4 has also been found to be a prognostic marker in multiple cancers including melanoma, colon cancer, leukemia, breast cancer, and gliomas [15,16]. Therefore, as CXCR4 is overexpressed in several human cancers, the blockade of CXCR4–CXCL12 interaction has been extensively investigated as a potential cancer therapeutic. Recently, we designed and synthesized two short flexible peptides, they both possess a CPC motif. Then we analyzed their structure by means of experimental and computational methods. Finally, we evaluated their interaction with CXCR4 by means of *in vitro* studies at 1 and 10 µM concentrations [15].

Hence, in this work we carried out biological tests to evaluate if PepE and PepK were able to: (i) interact with the CXCR4 receptor; (ii) inhibit CXCL12-induced migration; and (iii) reduce cAMP (Cyclic Adenosine Monophosphate) levels. Our data pointed out that PepE and PepK target the CXCR4 receptor by modulating the adenylate cyclase and they represent the starting point to design useful “*instruments*” for molecular recognition processes in which a flexible conformation is needed for the interaction with a specific target.

## 2. Results and Discussion

The two linear intrinsically disordered peptides PepK and PepE have been designed choosing a few amino acids with disorder propensity. A Lys residue has been added at *C*-terminal side of PepK and a Glu residue has been added at the *C*-terminal side of PepE. The conformational features of both peptides were studied in solution by CD, NMR and MD techniques.

### 2.1. Circular Dichroism

**Figure 1:**
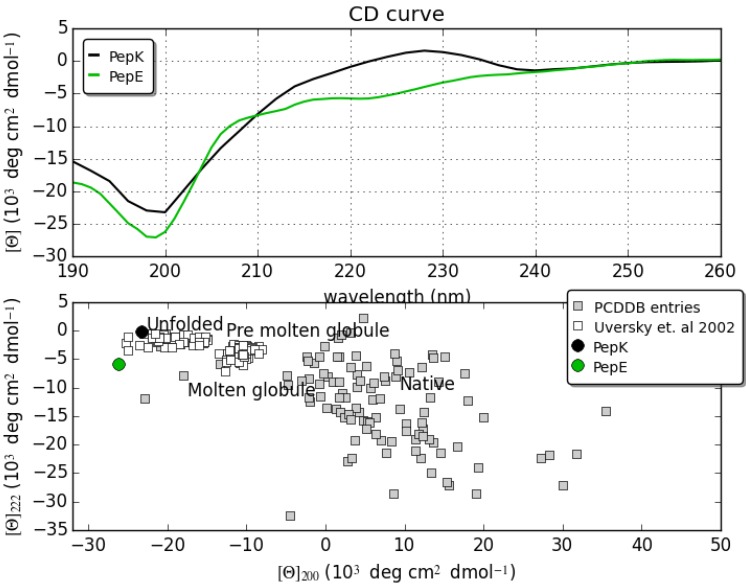
CD spectra of PepK and PepE (**upper** panel) and CD analysis by CAPITO tool (**lower** panel) where both peptides are indicated as unfolded peptides.

The secondary structure of PepE and PepK in PBS was studied by circular dichroism (CD) spectroscopy and analyzed by the CAPITO web server (http://capito.nmr.fli-leibniz.de). As expected for very short peptide sequences, both peptides did not show any tendency to fold under this experimental condition [17]. CD spectra (Figure 1) show a typical shape of an unordered structure with a negative band between 197 and 201 nm. This behavior is also confirmed by CAPITO analysis [18], where spectra values—such as mean residue ellipticity (θ)—at λ = 200 nm are plotted *versus* λ = 222 nm. In fact, both the peptides are located in unfolded regions. It is important to notice that the CD spectrum of PepK presents also a maximum centered at 225 nm that has been already associated to the presence of fluctuating Polyproline II (PII) helices by various authors in recent papers [13,19,20].

### 2.2. NMR Characterization

**Figure 2:**
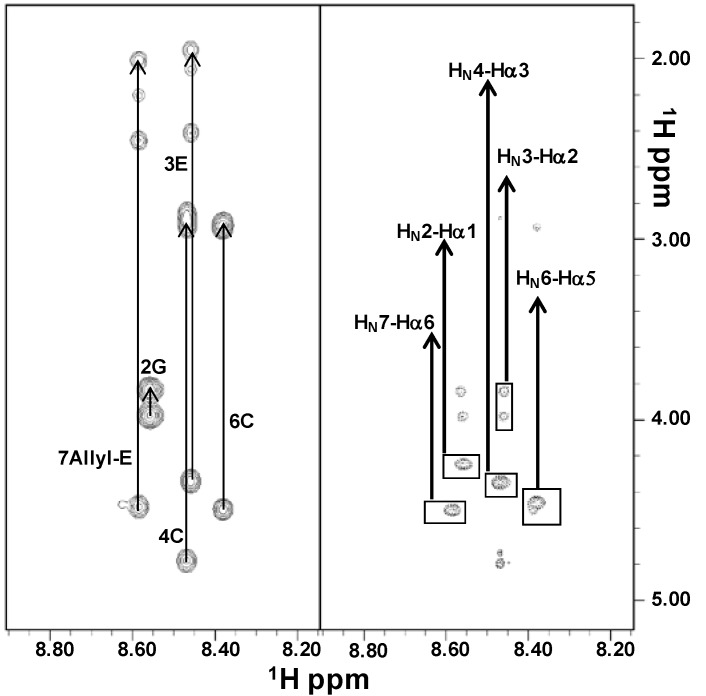
Comparison of 2D [^1^H, ^1^H] TOCSY (**left**) and ROESY (**right**) spectra of PepE in H_2_O/D_2_O (90/10). The H_N_-aliphatic protons correlation regions are shown in each panel; spin system assignments are indicated in the left side. In the right panel, sequential ROE contacts are highlighted by rectangles and the corresponding assignments are indicated.

**Figure 3:**
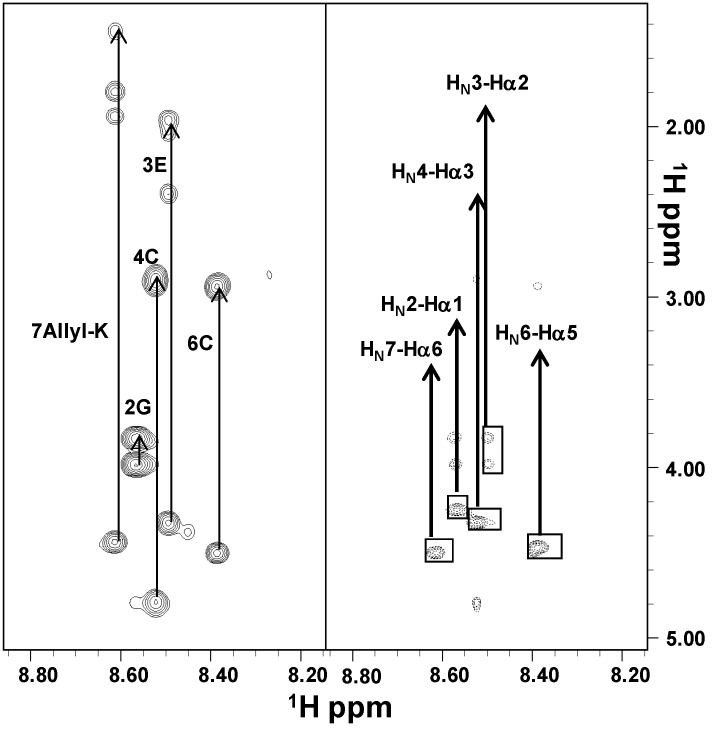
Comparison of 2D [^1^H, ^1^H] TOCSY (**left**) and ROESY 200 (**right**) spectra of PepK in H_2_O/D_2_O (90/10). The two sides of the figure show spectral regions containing H_N_/aliphatic protons correlations; spin system assignments are indicated in the TOCSY left panel. In the right panel, sequential ROE contacts are shown.

In aqueous solution, the poor spectral dispersion of the 1D [^1^H] spectra and the almost complete absence of signal in the 2D [^1^H, ^1^H] NOESY [21] experiments indicated that both peptides were very flexible (data not shown). However, complete proton resonance assignments were achieved by combined analysis of the 2D [^1^H, ^1^H] TOCSY [22] and ROESY [23] spectra (Figure 2 and Figure 3; Tables S1 and S2).

Moreover, we evaluated chemical shifts deviations of H_α_ protons from random coil values (Tables S3 and S4), which resulted in prevalence small and positive and characteristic of an extended disordered conformation [24]. The disorder state of both peptides was further strengthened by ROE patterns (Figure S1) which showed strong and sequential H_α_*^i^*–H_N_*^i+1^* contacts typical of flexible peptides [25]. Due to the proximity of chemical shifts between Cys4 H_α_ proton and water in both PepE and PepK, we could not clearly identify the configuration (*i.e.*, *cis* or *trans*) of the Cys4–Pro5 peptide bond.

### 2.3. Molecular Dynamics Simulations on the Two Peptides

**Figure 4:**
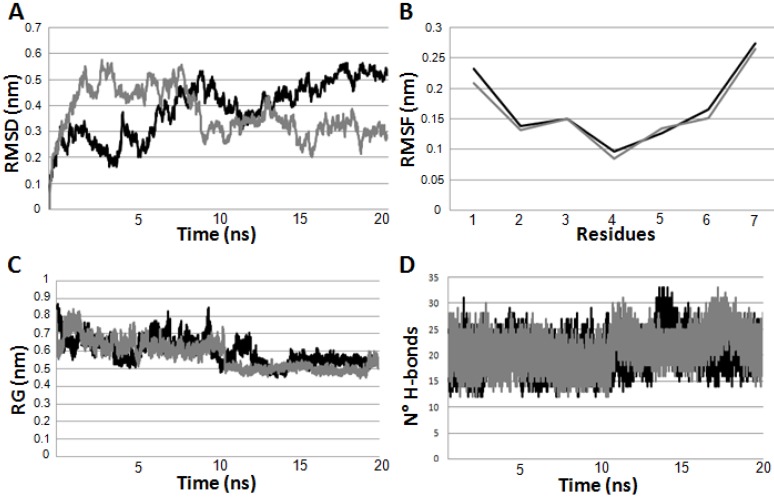
Analysis of the molecular dynamics simulations performed on PepE (in black) and PepK (in grey) at physiological pH in explicit solvent in terms of: (**A**) root mean square deviation (RMSD) plot; (**B**) root mean square fluctuation (RMSF); (**C**) gyration radius plot; and (**D**) H-bonds plot.

Molecular dynamics simulations were performed on the two peptides, which were linearly modeled as reported in the Material and Methods Section, at neutral pH. In Figure 4A, we show the plots of the Root Mean Square Deviation (RMSD), computed by overlapping the various structures during simulations in respect to the initial conformation. RMSD plots of PepK and PepE evidence high levels of fluctuation, thus suggesting that these peptides are flexible. On the one hand, this result is confirmed also by Root Mean Square Fluctuation (RMSF) plots where the residues located at *N*- and *C*-termini present higher values of RMSF (Figure 4B). Instead, the gyration radii decreased during the simulations (Figure 4C) reaching a value of 0.5 nm. This suggests an increasing compactness of the two peptides, which result as if they were stabilized by a certain number of H-bonds of main chain–main chain (MM), main chain–side chain (MS) and side chain–side chain (SS) types (Figure 4D). For this reason, the two peptides tended to become more compact and the radius of gyration decreased. According to the data obtained by CD and NMR measurements in aqueous solution, the analysis of the secondary structure evolution clearly showed the high flexibility of both peptides during the simulations, which makes difficult the formation of stable regular secondary structure elements, *i.e.*, helix and β-strand. Very flexible peptide structure are normally characterized by several conformers at equilibrium, hence they could be well described by a conformational ensemble. Having taken this property into proper account, we have firstly performed a cluster analysis to determine the groups of structures that share similar conformational features according to their RMSD values.

**Figure 5:**
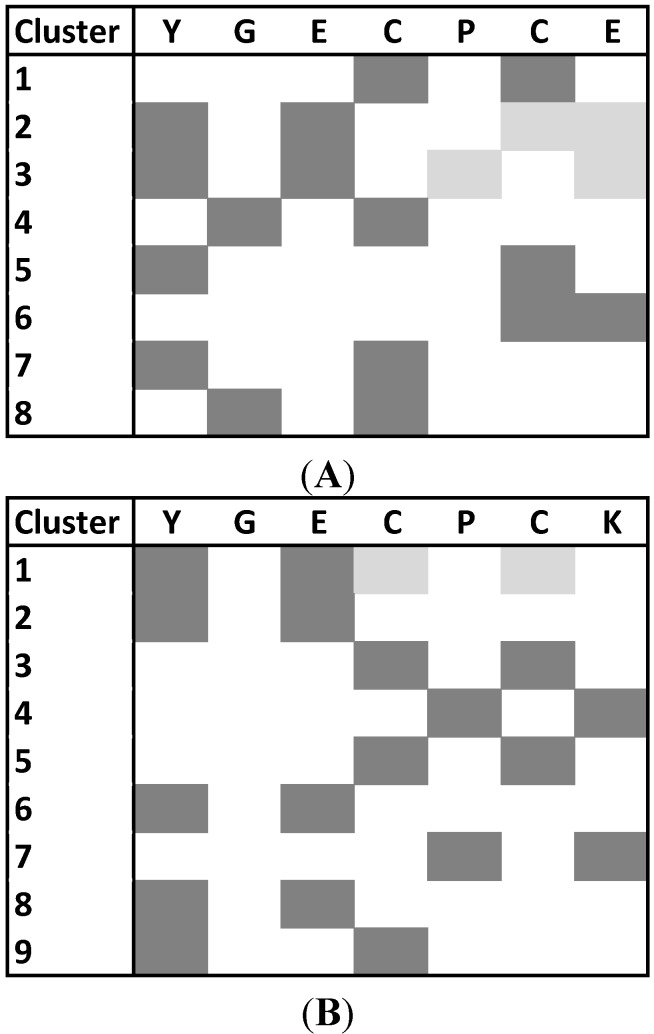
The map of MM H-bonds in the 8 and 9 clusters calculated for the PepE (**A**) and PepK (**B**), respectively, during MD simulations. We reported with the same colors the residues involved in the same MM H-bonds.

The number of the most populated clusters resulted as equal to 8 for PepE and 9 for PepK. These clusters are mainly stabilized by MM H-bonds (Figure 5) that involved the atomic groups of the peptide backbone. This structural organization was observed by Pappu *et al.* [26] for charged peptides: it closely resembles the organization of collapsed and slightly soluble globules.

Since CD spectra of PepK pointed out the presence of PII, we verified its possible occurrence in the most populated clusters by using the angular ranges following reference parameters for the angles: −110° ≤ φ ≤ −40° and 130° ≤ ψ ≤ 180° in the Ramachandran map [27]. No residues in PII were present in eight clusters of PepE whereas in 5 out of 9 clusters of PepK we identified residues in PII (Figure S2), according to CD analysis.

It is well known that the peptide dynamics depend on the surrounding solvent, which mediates interactions among residues. Therefore, we have analyzed the total number of water molecules in the system and their possible role [28]: the simulation box for PepE and PepK contained 2956 and 2611 water molecules, respectively. The total average number of water molecules that formed H-bonds resulted slightly higher for PepE if compared to PepK: 33 and 30 in PepE and PepK, respectively. This trend could be due to the different amino acid sequence of the two peptides; in fact PepK has got two oppositely charged residues in 3 and 7 positions, hence it may assume a more compact structure. The majority of H-bonds (indicatively 60%) for both peptides resulted to involve the peptide backbone, being of main chain—water oxygen type (MH) or of the water oxygen and main chain type (HM). A detailed analysis of the residues involved in H-bonds with water molecules pointed out that the residues in positions 1, 3 and 7 (Y, E and E in PepE and Y, E and K in PepK) have developed a higher number of H-bonds with water molecules than the residues located in the other four positions (Table S5). Furthermore, the average number of H-bonds developed by E and K with water molecules was lower than the average number of the H-bonds developed by E and E with water molecules in PepE. Hence, these results confirmed that PepE tended to assume more extended conformations being that it contains two negatively charged residues that could allow large interactions with water molecules.

Generally, the data evidence that the structure of both peptides is very flexible, and can be dynamically stabilized by a network of MM H-bonds. Hence, their structures can be adequately represented as conformational ensembles characterized by fluctuating irregular secondary structures. In fact, the peptides do not assume stable and fixed conformations: they go continuously from one cluster to another. As the high flexibility of peptides did not allow us to calculate a 3D model based on NMR parameters, the MD was very useful to understand how these peptides can move as well as to understand that the best conformers needed to be put together to have a clear representation. In particular, the two peptides do not explore the full space, but rather, the rapid interchange among the flexible conformers induce quick changes between clusters. This is in line with the data shown in Figure 4B where it is visible that the more flexible residues are located in the terminal ends.

### 2.4. Functional Characterization on CXCR4 Receptor

We have designed a short disordered peptide the containing CPC motif and we have analyzed its binding capability on the CXCR4 receptor finding a behavior as ligand [15]. Hence, we performed PepK and PepE functional characterization on the same chemokine receptor. In order to assess the inhibitory capability of PepK and PepE on CXCR4 function, we have evaluated peptides binding to the receptor through an indirect binding assay; then, in addition we have assessed the inhibition of CXCL12-induced migration and evaluated the activity of the adenylate cyclase function. The results were compared to Plerixafor (known as AMD3100) that is used as a clinical antagonist of CXCR4. The treatment with plerixafor plus Granulocyte-colony stimulating factor (G-CSF) induced the mobilization of hematopoietic precursors from bone marrow, allowing a more efficient recovery of the CD34 stem cells necessary for autologous stem cell transplantation [29].

The binding of the two peptides to CXCR4 was evaluated in CCRF-CEM cells using a CXCR4 12G5 monoclonal antibody as previously described [30]. PepK and PepE were evaluated at concentrations similar to the peptidic antagonists recently developed [16,30]. Indeed, we have tested only two peptide concentrations (1 and 10 µM), which are matching the ones previously used in CXCR4 binding assays. As shown in Figure S3, PepK and PepE did not displace the 12G5 antibody compared to AMD3100, which strongly reduced antibody binding to CXCR4 (Figure S3). These results may be explained considering the different binding sites on AMD3100/12G5 antibody and the high flexibility of the two disordered peptides. Moreover, cAMP and migration assays were carried out as well in order to evaluate the peptide ability to inhibit the activation of CXCR4 mediated intracellular pathways.

CXCR4 is a G-Protein coupled receptor, which, as result of the binding to CXCL12, inhibits through the G-protein the adenylate cyclase activity and the inhibition of cAMP production as “second messenger” [31]. In Figure S4, preliminary biological results are summarized. The results underline that in the presence of CXCL12 (100 ng/mL) and Forskolin (1 µM): (i) PepE determines a dose dependent increase of the cAMP level of 20% at the lower dose (1 μM) and 65% at the higher dose (10 μM); (ii) PepK increases cAMP but the effect is not dose-dependent; and (iii) AMD3100 has activities comparable or even less efficient than PepE. Hence, PepK and PepE result to modulate the adenylate cyclase at equal or higher levels as AMD3100.

Finally, the migration was performed (Figure S5) and, both PepK and PepE reduced CCRF-CEM cells migration towards CXCL12 although less effectively than AMD3100. In particular, PepE reduces the migration index from 1.75- to 1.35-fold and PepK from 1.75- to 1.55-fold, whereas AMD3100 from 1.75- to 1.15-fold.

## 3. Experimental Section

Protected fluorenylmethoxycarbonyl (Fmoc)N^α^-Fmoc-amino acid derivatives, 2-chlorotrityl chloride resin pre-loaded with Fmoc-Glu-OAllyl and Fmoc-Lys-OAllyl and coupling reagents were purchased from Novabiochem (Schwalbach, Germany) and Iris Biotech GMBH (Marktredwitz, Germany). All other chemicals were commercially available by Sigma–Aldrich (Milwaukee, WI, USA) and Acros (Geel, Belgium). The purity of products was determined by analytic reverse-phase HPLC using a VWR Hitachi instrument (Radnor, PA, USA) equipped with a L-2450 auto sampler, two L-2130 pumps, a Satisfaction RP18-AE column (5 μm, 250 × 4.6 mm) and a L-2450 diode array detector, at a flow rate of 0.8 mL/min.

The compounds were purified by preparative reverse-phase HPLC using a VWR LaPrep system consisted of a P202 injector, two P110 pumps, a Satisfaction RP18-AB C18 column (5 μm, 250 × 20 mm) and a P314 UV detector, at a flow rate of 10 mL/min.

The following eluents were used in a gradient mode: (A) 0.1% trifluoroacetic acid (TFA) in H_2_O/CH_3_CN (95/5) and (B) 0.1% TFA in CH_3_CN/H_2_O (95/5). Water was of Milli-Q quality and was obtained after filtration of distilled water through a Milli-Q^®^ cartridge system (Millipore Co., Billerica, MA, USA). CH_3_CN and TFA were of HPLC use quality. Degassing of solvents was performed using argon bubbling. Gradient used for the analytic and preparative RP-HPLC was respectively from 0% B to 100% B in 15 min and from 0% to 70% B in 70 min; UV detection at 214 nm. CH_3_CN was evaporated and the aqueous solution was freeze-dried to give purified PepK and PepE as white solids (68% and 54% yield, respectively).

Peptide identity was confirmed by mass spectrometry analyses performed on an Ultraflex III TOF/TOF system (Bruker Daltonics, Bremen, Germany), equipped with 200 Hz smart beam laser (355 nm) and operating in reflectron positive ion mode. Mass spectra were acquired over the *m*/*z* range 450–5000 by accumulating data from 1000 laser shots for each spectrum. The instrumental conditions employed to analyze molecular species were the following: ion source 1: 25.08 kV; ion source 2: 21.98 kV, lens: 11.03 kV, pulsed ion extraction: 30 ns, reflector: 26.39 kV, reflector 2: 13.79 kV. Matrix suppression was activated by deflection mode: suppression up to 450 Da. Mass calibration was performed for each samples in range of ~700–3200 Da with a peptide calibration mixture (8206195, Peptide Calibration Standard, Bruker Daltonics). The instrument was controlled using Bruker’s flexControl 3.4 software and mass spectra were analyzed in Bruker’s FlexAnalysis 3.4 software.

### 3.1. Synthesis

PepK (sequence: YGECPCK-OAllyl) and PepE (sequence: YGECPCK-OAllyl) were synthesized by Fmoc standard chemistry protocol on 2-chlorotrityl chloride resin pre-loaded with the side chain of a Fmoc-Lys-OAllyl and a Fmoc-Glu-OAllyl, respectively (0.24 mmol scale).

Swelling and Fmoc-deprotection: Swelling and Fmoc-deprotection steps were performed by treating the resin for 30 min in dichlorometane (DCM) and in dimethylformamide (DMF)/Piperidine (Pip) (80/20, *v*/*v*) solution, respectively.

Washing: Washing steps after coupling and deprotection steps were performed by treating resin in DMF (3 × 1 min), MeOH (1 × 1 min), and DCM (3 × 1 min), successively.

Amino acid coupling: The resin was immersed and mixed in a DMF solution containing the Fmoc-protected amino acid (3 eq), *O*-benzotriazole-tetramethyl-uronium-hexafluoro-phosphate (HBTU) (3 eq), and diisopropylethylamine (DIEA) (6 eq) for 2 h at room temperature.

Peptide cleavage: The peptide was cleaved by immersing the resin in TFA/H_2_O/triisopropylsilane (TIS) (30 mL, 95/2.5/2.5, *v*/*v*/*v*) for 3 h. The cleavage cocktail was filtered from the resin, peptides were precipitated with ice-cold diethyl ether, centrifuged, and decanted. Precipitation, centrifugation, and decantation operations were repeated twice. The resulting white solid was dissolved in water (10 mL) and freeze-dried to give a white powder that was analyzed for purity and purified by preparative RP-HPLC, using the specified conditions.

### 3.2. Circular Dichroism Measurement

Far-UV CD spectra were recorded from 190 to 260 nm on a Jasco J-810 spectropolarimeter (JASCO Inc., Easton, MD, USA) equipped with a NesLab RTE111 thermal controller unit using a 1 mm quartz cell at 25 °C. Circular dichroism measurements were carried out on lyophilized peptides dissolved in 10 mM phosphate buffer at pH 7.4 M at a peptide concentration of 5 × 10^−6^ M. Other experimental settings were: scan speed, 10 nm·min^−1^; sensitivity, 50 mdeg; time constant, 16 s; bandwidth, 1 nm. Each spectrum was obtained averaging three scans, and by subtracting contributions from other species in solution and converting the signal to mean residue ellipticity in units of deg·cm^2^·dmol^−1^·res^−1^.

### 3.3. NMR Analysis

NMR spectra were recorded at 25 °C on a Varian Unity Inova 600 MHz spectrometer (Varian Inc., Palo Alto, CA, USA) provided with a cold probe. The process of proton resonance assignments was carried out with a canonical protocol [25] based on analysis of the following two dimensional [1H, 1H] spectra: TOCSY (Total Correlation Spectroscopy) (70 ms mixing time) [22], DQFCOSY (Double Quantum Filter Correlation Spectroscopy) [32], NOESY (Nuclear Overhauser Enhancement Spectroscopy) [21] (300 ms mixing time) and ROESY (Rotating frame Overhauser Enhancement Spectroscopy) [23] (200 ms mixing time). Chemical shifts were referenced with respect to the TSP (trimethylsilyl-3-propionic acid sodium salt-d4, 99% d, (Armar Scientific, Döttingen, Switzerland) signal at 0.0 ppm.

1D spectra were acquired with a relaxation delay of 1 s and 32–128 scans. 2D experiments were generally acquired with 32–64 scans, 128–256 FIDs in *t*_1_, 1024 or 2048 data points in *t*_2_. The DPFGSE (Double Pulsed Field Gradient Selective Echo) sequence [33] was used to suppress water signal. Spectra were processed with the Varian software VNMRJ 1.1D (Varian by Agilent Technologies, Milan, Italy) and analyzed with the NEASY [34] program that is included in the CARA (Computer Aided Resonance Assignment) software package (http://www.nmr.ch/).

### 3.4. Molecular Modeling and Dynamics Simulations

PepK and PepE have been built by using the Builder module in InsightII and then subjected to molecular dynamics (MD) simulations performed with the GROMACS software package (v3.3.1) [35]. In detail, each peptide was put in a cubic box filled with SPC216 water molecules, and GROMOS43a1 was selected as force field because it is commonly used as force field for MD simulations on peptides and our group has already used it in other previous papers in which we reported some conformational studies on different peptides where the results obtained from MD simulations proved to be in excellent agreement with CD and/or NMR studies [13,15,16]. In order to optimize the systems, the peptides were previously subjected to energy minimization and position restraints cycles. The simulations were carried out with periodic boundary conditions by adding chloride ions so that the net electrostatic charge of the system was zero. The bond lengths were constrained by the linear constraint solver algorithm. Particle mesh Ewald algorithm was used for the electrostatic interactions with a cutoff of 0.9 nm, according to our recent papers [13,15,36]. All simulations were run for 20 ns at neutral pH and room temperature (300 K) coupling to the system an external bath. GROMACS routines RMSD and RMSF, gyration radius, number of H-bonds and secondary structure evolution were utilized to check the trajectories and the quality of the simulations. Additionally, on the basis of the distances between structures, like RMSD values, we founded a set of clusters that reflects the range of conformations accessible and the relative weight of each of these by using a clustering algorithm implemented in GROMACS as reported also in our recent papers [13,16]. The presence of putative H-bonds between the residues and with water molecules was evaluated by Hbplus [37].

### 3.5. Migration Assay

CCRF-CEM cells migration was assayed in 24-well Transwell chambers (Corning Inc., Corning, NY, USA) using inserts with an 8-μm pore membrane. Membranes were precoated with collagen (human collagen type I/III) and fibronectin (20 mg/mL each). CCRF-CEM cells were placed in the upper chamber (1 × 10^5^ cells/well) in RPMI containing 1% BSA (migration media). Cells were pre-incubated for 45 min with CXCR4 antagonist and allowed to migrate toward 100 ng/mL CXCL12 in the lower chamber. After 16 h incubation, migrated cells were collected from the lower chamber and counted. The migration index was defined as the ratio between migrating cells in the experimental group and migrated cells in the control group.

### 3.6. Binding Assay

PepK and PepE binding to CXCR4 was evaluatedas previously described [30]. Briefly 2.5 × 10^5^ CCRF–CEM cells were pre-incubated with 10 μM antagonist peptides in binding buffer (PBS 1× plus 0.2% BSA and 0.1% NaN_3_) for 1 h at 37 °C, 5% CO_2_ and then labeled for 45 min with anti-CXCR4 PE-antibody (FAB170P, clone 12G5, R&D Systems, Minneapolis, MN, USA). Cells were washed in PBS and analyzed by FACS Canto II cytofluorimeter (Becton Dickinson Immunocytometry Systems, Mountain View, CA, USA).

### 3.7. cAMP Assay

CCRF-CEM cells (1 × 10^6^) were incubated in presence of PepK, PepE or Plerixafor (known as AMD3100) being a CXCR4 antagonist that has provided proof of concept for inhibition of the pathway [31] at different concentrations (1 and 10 μM) in combination with forskolin (F) (1 μM) for 20 min, followed by stimulation with CXCL12 (100 ng/mL) for 10 min. Controls include cells stimulated with CXCL12 and forskolin or forskolin alone in absence of anti-CXCR4 inhibitors. Cells are harvested and lysed with 0.1 M HCl and cAMP levels was assayed using a direct competitive enzyme immunoassay (BioVision Incorporated, Milpitas, CA, USA) according to manufacture instructions.

## 4. Conclusions

As CXCR4 is overexpressed in several human cancers, the blockade of CXCR4–CXCL12 interaction represents from many years an interesting drug-target [15]. Moreover, the plerixafor (previously known as AMD3100) is the most clinically advanced compound even if it showed cardiotoxicity, as reported in its clinical trial against HIV [38]. Therefore, this suggests the need to develop new antagonists able to block, without any other compliance, the binding between CXCL12 and CXCR4. In this work we have described the structural preferences in solution of two synthetic linear peptides containing a few amino acids with disorder propensity, and an allylic ester group at the *C*-terminal end. NMR and CD solution studies, revealed the absence of regular secondary structure elements thus confirming the natively disordered nature of these peptides. MD simulations evidenced that, even if they are flexible, they are stabilized by a network of transient and dynamic intra-molecular H-bonds of MM type and by interactions with water molecules. In details, NMR and computational studies showed the flexibility of PepE and PepK, their inability to adopt a single ordered conformation and, hence, the need to represent them by conformational ensembles characterized by the presence of transient and dynamic MM H-bonds.

Moreover, we have performed a functional characterization of the two peptides by investigating (a) their ability to interact with the CXCR4 receptor; (b) the inhibition of CXCL12-induced migration and (c) the reduction of cAMP levels. Generally, we pointed out that PepK and PepE result to modulate the adenylate cyclase as much or more as AMD3100, *i.e.*, the best characterized CXCR4 inhibitor. These results indicate that the short flexible peptides could efficiently target the CXCR4 receptor.

This preliminary evidence suggests that the PepK and PepE interact with CXCR4 and they represent a potential starting system able to be converted to more efficient *in vitro* and *in vivo* antagonists of CXCR4.

## Supplementary Materials

Supplementary materials are available at http://www.mdpi.com/1422-0067/16/06/12159/s1.
