# Supplementary material for: Conformational Ensembles Explored Dynamically from Disordered Peptides Targeting Chemokine Receptor CXCR4
Source: Int J Mol Sci. 2015 May 28;16(6):12159–73. doi: 10.3390/ijms160612159 (PMC4490436; doi:10.3390/ijms160612159)
Supplement: Supplementary file 1 [file ijms-16-12159-s001.pdf]

# Supplementary Information

**Table S1.** Chemical shifts (ppm) of PepE evaluated at 600 MHz and 298 K.

| **Residue** | **$H_N$** | **$H_\alpha$** | **$H_\beta$** | **$H_\gamma$** | **Others** |
|---|---|---|---|---|---|
| ♦ 1Y | | 4.23 | 3.13 | | Hδ 7.16<br>Hε 6.88 |
| 2G | 8.56 | 3.98<br>3.82 | | | |
| 3E | 8.46 | 4.33 | 2.08<br>1.97 | 2.42 | |
| 4C | 8.47 | 4.77 | 2.94<br>2.87 | | |
| 5P | | 4.45 | 2.30 | 2.03<br>1.96 | Hδ 3.77 |
| 6C | 8.38 | 4.49 | 2.94 | | |
| 7Allyl-E | 8.58 | 4.48 | 2.22<br>2.02 | 2.46 | Allyl group 5.96, 5.36, 5.30, 4.75 |

♦ Amino acids are indicated by the one letter codes preceded by sequence number.

**Table S2.** Chemical shifts (ppm) of PepK evaluated at 600 MHz and 298 K.

| **Residue** | **$H_N$** | **$H_\alpha$** | **$H_\beta$** | **$H_\gamma$** | **Others** |
|---|---|---|---|---|---|
| ♦ 1Y | | 4.24 | 3.13 | | Hδ 7.16, 7.11<br>Hε 6.88, 6.84 |
| 2G | 8.57 | 3.98<br>3.82 | | | |
| 3E | 8.50 | 4.32 | 2.04<br>1.96 | 2.39 | |
| 4C | 8.53 | 4.78 | 2.91 | | |
| 5P | | 4.45 | 2.30 | 2.02<br>1.95 | Hδ 3.78 |
| 6C | 8.39 | 4.49 | 2.93 | | |
| 7Allyl-K | 8.61 | 4.42 | 1.94<br>1.80 | 1.45 | Hε 2.99<br>Hδ 1.68<br>Hζ 7.53<br>Allyl 5.95, 5.34, 5.28, 4.67 |

♦ Amino acids are indicated by the one letter codes preceded by sequence number.

**Table S3.** Chemical shift deviations of $H_\alpha$ protons from random coil values (CSD) for PepE [24]. The CSD value for Allyl-E (residue number 7) is not shown due to the lack of a tabulated random coil value for the modified amino-acid. For Gly average $H_\alpha$ chemical shift values were taken into accounts.

| Residue | $H\alpha_{obs}$ | $H\alpha_{randomcoil}$ | CSD ($H\alpha_{obs} - H\alpha_{randomcoil}$) |
|---|---|---|---|
| ♦ 1Y | 4.23 | 4.43 | −0.2 |
| 2G | 3.98 | 4.11 | 0.02 |
| | 3.82 | 3.65 | |
| 3E | 4.33 | 4.24 | 0.09 |
| 4C | 4.77 | 4.52 | 0.25 |
| 5P | 4.45 | 4.33 | 0.12 |
| 6C | 4.49 | 4.52 | −0.03 |
| 7Allyl-E | 4.48 | – | – |

♦ Amino acids are indicated by the one letter codes preceded by sequence number.

**Table S4.** CSD (*i.e.*, $H\alpha_{obs} - H\alpha_{randomcoil}$) of PepK.

| Residue | $H\alpha_{obs}$ | $H\alpha_{randomcoil}$ | CSD ($H\alpha_{obs} - H\alpha_{randomcoil}$) |
|---|---|---|---|
| ♦ 1Y | 4.24 | 4.43 | −0.19 |
| 2G | 3.98 | 4.11 | 0.02 |
| | 3.82 | 3.65 | |
| 3E | 4.32 | 4.24 | 0.08 |
| 4C | 4.78 | 4.52 | 0.26 |
| 5P | 4.45 | 4.33 | 0.12 |
| 6C | 4.49 | 4.52 | −0.03 |
| 7Allyl-K | 4.42 | – | – |

♦ Amino acids are indicated by the one letter codes preceded by sequence number.

**Table S5.** The average number of H-bonds that each residue in PepE and PepK formed with water molecules during MD simulations.

| Residue | PepE | PepK |
|---|---|---|
| Y | 7 | 8 |
| G | 3 | 2 |
| E | 9 | 6 |
| C | 2 | 1 |
| P | 1 | 3 |
| C | 2 | 4 |
| E or K | 9 | 6 |

Amino acids are indicated by the one letter codes.

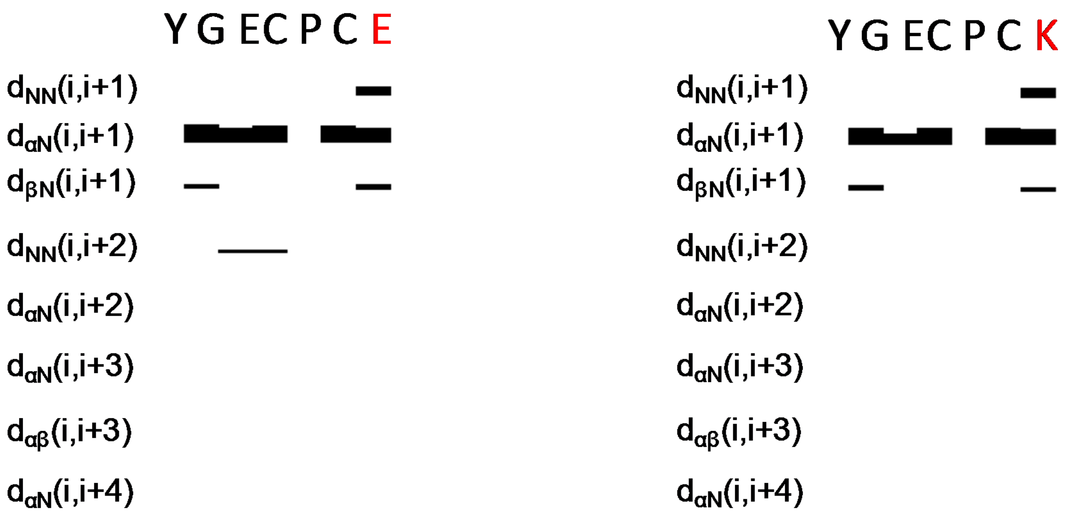


**Figure S1.** ROE diagrams for PepE (**left**) and PepK (**right**) in $H_2O/D_2O$ (90/10). Peptide sequences are reported on the top; "$d_{lm}$ ($i$, $i + x$)" indicates a contact between the Hl and Hm protons in the $i$ and $i + x$ residue respectively. The allyl-containing amino acids are highlighted in red.

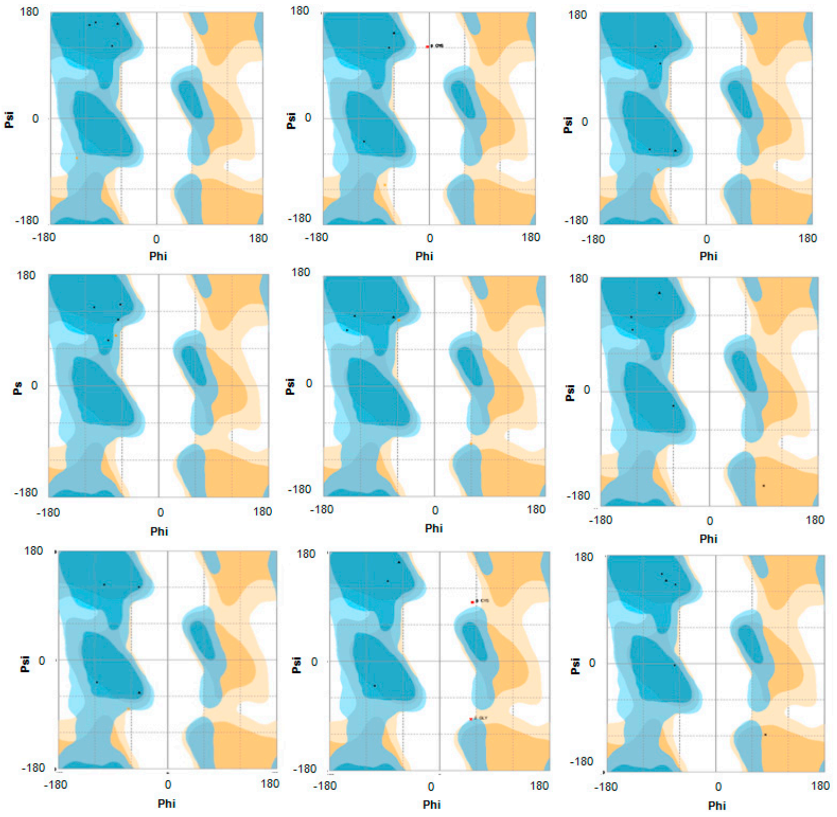


(**A**)

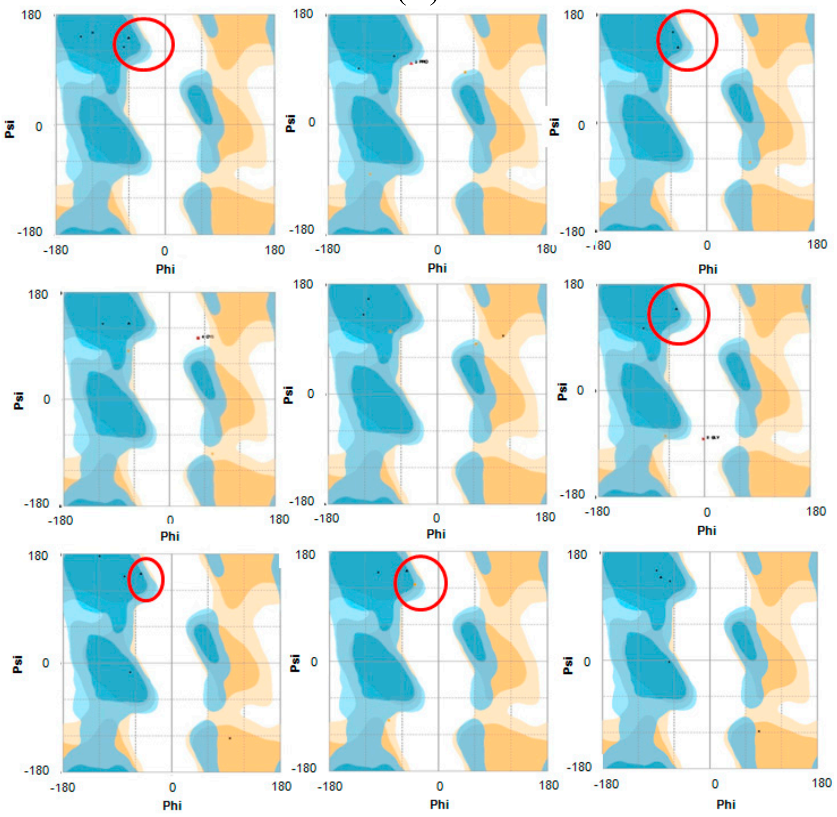


(**B**)

**Figure S2.** Ramachandran Plot for nine and eight clusters obtained for PepE (**A**) and PepK (**B**), respectively. We indicate by red circle the residues present in the polyproline II region.

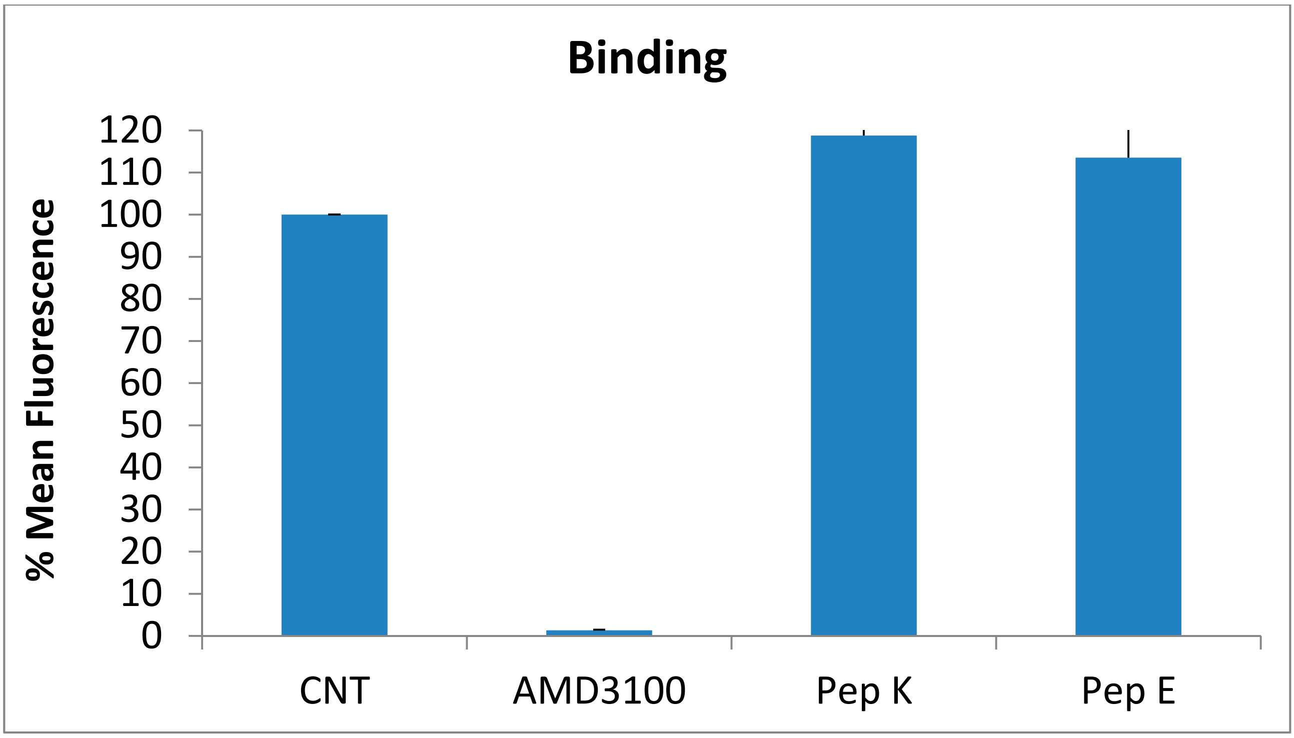


**Figure S3.** Binding of peptides to CXCR4. The binding was evaluated indirectly through flow cytometry. In particular, histograms indicate the fluorescence percentage for CXCR4 antibody (CNT), AMD3100, PepK and PepE.

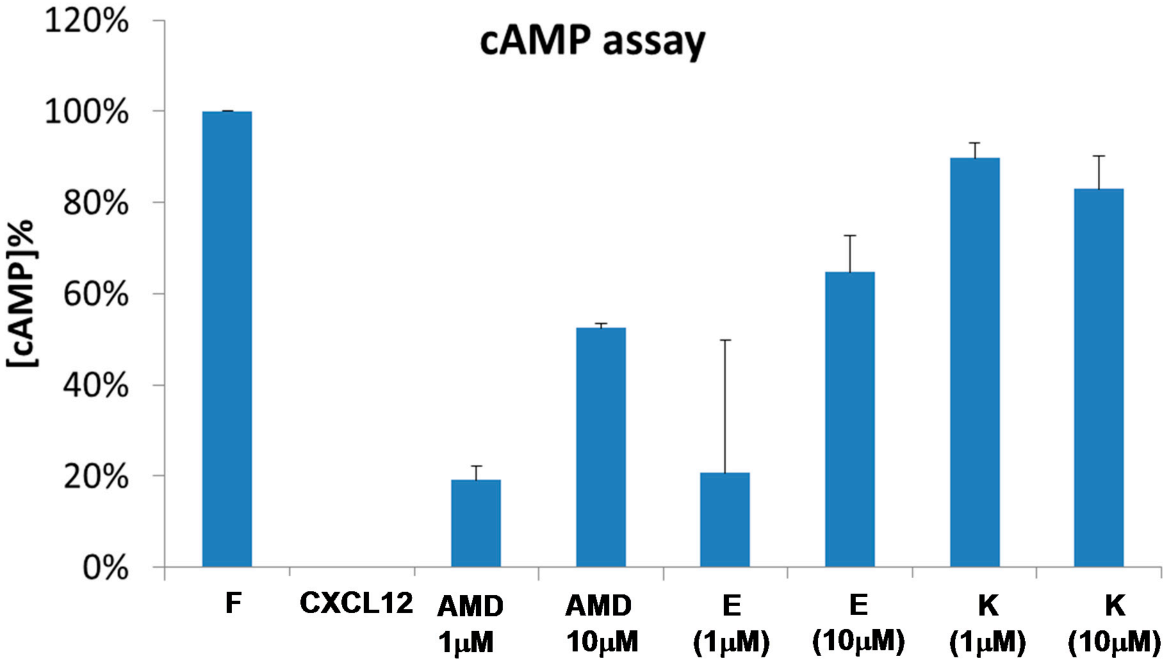


**Figure S4.** Comparison of inhibition of cAMP modulation by PepE, PepK and AMD3100 at 1 and 10 μM. Briefly, cells were incubated in the presence of AMD3100, PepE or PepK in the presence of CXCL12 plus Forskolin. CXCL12 100 ng/mL: maximal Gi protein activity; Forskolin 1 μM: maximal adenylate cyclase activity.

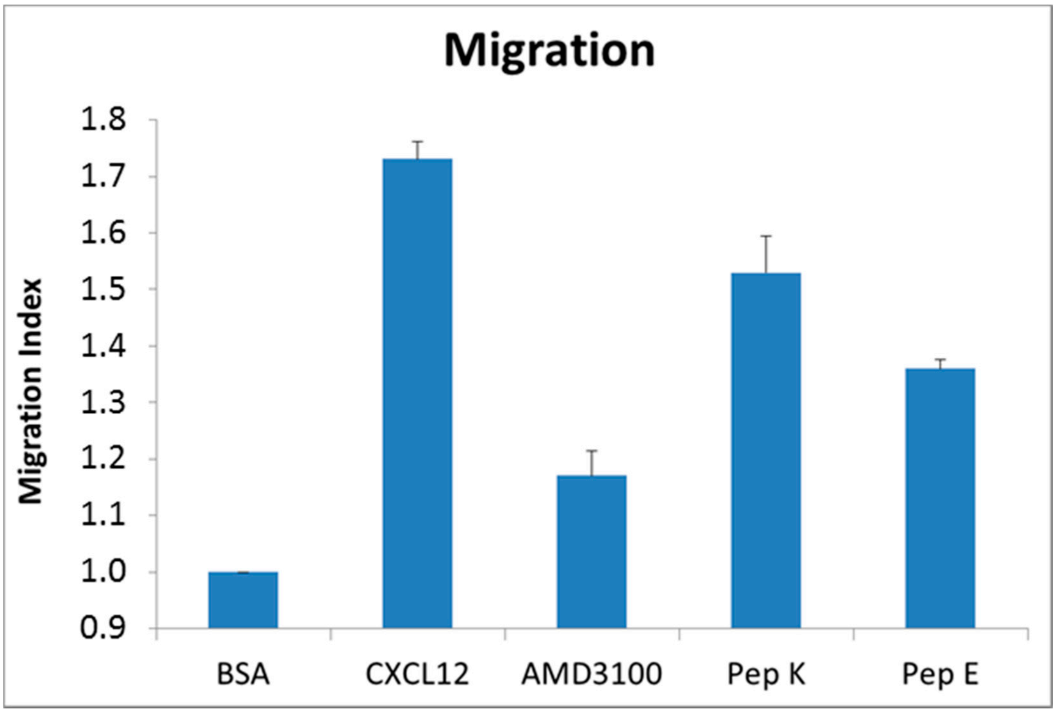


**Figure S5.** Migration assays. We report the migration index relative to migration in presence of BSA alone, and of CXCL12, AMD3100, PepK and PepE.
